# BMI modifies HDL-C effects on coronary artery bypass grafting outcomes

**DOI:** 10.1186/s12944-022-01739-2

**Published:** 2022-11-29

**Authors:** Malihe Rezaee, Aida Fallahzadeh, Ali Sheikhy, Mana Jameie, Amir Hossein Behnoush, Mina Pashang, Masih Tajdini, Hamed Tavolinejad, Farzad Masoudkabir, Soheil Mansourian, Shahram Momtahen, Hossein Ahmadi Tafti, Kaveh Hosseini

**Affiliations:** 1 Tehran Heart Center, Cardiovascular Diseases Research Institute, Tehran University of Medical Sciences, Tehran, Iran; 2 Cardiac Primary Prevention Research Center, Cardiovascular Diseases Research Institute, Tehran University of Medical Sciences, Tehran, Iran; 3 Medical Student Research Committee, School of Medicine, Shahid Beheshti University of Medical Sciences, Tehran, Iran; 4 Endocrinology and Metabolism Population Sciences Institute, Non-Communicable Disease Research Center, Tehran University of Medical Sciences, Tehran, Iran; 5 Cardiology Department, Tehran Heart Center, Tehran University of Medical Sciences, North Karegar Ave, P.O. Box: 1411713138, Tehran, Iran

**Keywords:** HDL-C, BMI, CABG, Outcome, Nonlinear relationship

## Abstract

**Background:**

Despite the recognized implications of high-density lipoprotein cholesterol (HDL-C) in cardiovascular diseases, the role of body mass index (BMI) in HDL-C association with cardiovascular outcomes remains unclear. This study investigated the possible modifying implications of BMI on the correlation between HDL-C and coronary artery bypass grafting (CABG) outcomes.

**Methods:**

The present cohort included isolated CABG patients (median follow-up: 76.58 [75.79–77.38] months). The participants were classified into three groups: 18.5 ≤ BMI < 25 (normal), 25 ≤ BMI < 30 (overweight), and 30 ≤ BMI < 35 (obese) kg/m^2^. Cox proportional hazard models (CPHs) and restricted cubic splines (RCSs) were applied to evaluate the relationship between HDL-C and all-cause mortality as well as major adverse cardio-cerebrovascular events (MACCEs) in different BMI categories.

**Results:**

This study enrolled a total of 15,639 patients. Considering the final Cox analysis among the normal and overweight groups, HDL-C ≥ 60 was a significant protective factor compared to 40 < HDL-C < 60 for all-cause mortality (adjusted hazard ratio (aHR): 0.47, *P*: 0.027; and aHR: 0.64, *P*: 0.007, respectively). However, the protective effect of HDL-C ≥ 60 was no longer observed among patients with 30 ≤ BMI < 35 (aHR: 1.16, *P* = 0.668). RCS trend analyses recapitulated these findings; among 30 ≤ BMI < 35, no uniform inverse linear association was observed; after approximately HDL-C≈55, its increase was no longer associated with reduced mortality risk. RCS analyses on MACCE revealed a plateau effect followed by a modest rise in overweight and obese patients from HDL-C = 40 onward (nonlinear association).

**Conclusions:**

Very high HDL-C (≥ 60 mg/dL) was not related to better outcomes among obese CABG patients. Furthermore, HDL-C was related to the post-CABG outcomes in a nonlinear manner, and the magnitude of its effects also differed across BMI subgroups.

**Supplementary Information:**

The online version contains supplementary material available at 10.1186/s12944-022-01739-2.

## Background

Coronary artery disease (CAD) is a major global challenge and the third leading cause of mortality worldwide, claiming approximately 17.8 million lives annually [1]. Coronary artery bypass grafting (CABG) surgery is the gold standard for severe multivessel CAD [2]. Following CABG, risk factor management is the mainstay of secondary prevention [3].

Over the past years, several studies have illustrated an inverse relationship between high-density lipoprotein cholesterol (HDL-C) levels and CAD as well as major adverse cerebro-cardiovascular events (MACCEs). In practical guidelines, low serum HDL-C levels are considered an indispensable risk factor for CAD incidence and a risk predictor of cardiovascular outcomes [4–7]. Nevertheless, this simple linear correlation has been challenged in previous studies [8–10].

In addition to the lipid profile, obesity has a critical and independent role in the development and progression of CAD, with both direct and indirect effects on other risk factors [11, 12]. A nonlinear, U-shaped correlation between BMI and mortality in patients with acute coronary syndrome (ACS) has been reported, exhibiting lower mortality in overweight or mild-moderate obese patients paradoxically [13, 14]. However, some other studies have challenged this paradoxical U-shaped association [15].

Several studies have indicated that elevated body mass index (BMI) could be related to reduced HDL-C concentrations. Furthermore, in the context of obesity, the morphology and function of adipose tissue are changed, which can affect HDL-C functioning [16, 17]. Given the conflicting relationship between HDL-C and CABG outcomes, as well as the simultaneous intricate association of BMI with both of them, the present study evaluated the effects of serum HDL-C levels among different ranges of BMI on MACCE and all-cause mortality after CABG.

## Methods

### Design

This registry-based prospective study was performed at Tehran Heart Center (THC) [18], evaluating CABG patients between January 2006 and December 2016. The study was performed according to the Declaration of Helsinki. The ethics committee of Tehran Heart Center approved the study (IR.TUMS.VCR.REC.1400.09.12). As this study did not meet the criteria for informed consent, an “informed consent waiver” was attained from the ethics board. The study complies with the STROBE checklist for cohort studies. Details on the checklist can be found in the supplementary files.

### Population

All patients undergoing isolated CABG at THC were included, while patients with a BMI > 35 (morbidly obese) and those with a BMI < 18.5 were excluded. Furthermore, those with a history of presurgery myocardial infarction (MI) with > 21-day MI-CABG interval, those with missing data on HDL-C or BMI or any other baseline variables at baseline, and lost-to-follow-up patients were excluded from final analyses. Finally, 15,639 patients were included.

### Baseline assessment

Trained staff measured the heights and weights of the study population using standard guidelines. BMI was calculated as follows: Weight (kg)/height2 (m^2^). According to weight management guidelines, the patients were classified into three groups: 18.5 ≤ BMI < 25 (normal weight), 25 ≤ BMI < 30 (overweight), and 30 ≤ BMI < 35 (obese).

Baseline characteristics of the patients, including age, smoking status, past medical history (previous MI (< 24 h, 1–7 days, and 8–21 days), cerebrovascular accident (CVA), carotid stenosis, positive family history of IHD, chronic obstructive pulmonary disease (COPD)), and drug history, were obtained. Furthermore, echocardiographic left ventricular ejection fraction (LVEF), estimated glomerular filtration rate (eGFR), HDL-C (mg/dL) and low-density lipoprotein cholesterol (LDL-C) (mg/dL) levels were measured preoperatively. Variable definitions were in line with previous studies on this population [19].

### Follow-up and study endpoints

The patients were followed for a median of 76.58 [75.79–77.38] months, equivalent to 95,539.52 person-years. Follow-ups were in person or by telephone interviews (for patients who were unable or unwilling to attend the clinic). The study endpoints, including MACCE (including all-cause mortality, stroke, acute coronary syndrome, and revascularization) and mortality, were recorded in each follow-up session.

### Statistical analyses

Mean (standard deviation (SD)) and median [25^th^ and 75^th^ percentiles] were used to present normally and nonnormally distributed continuous variables, respectively. Normality was assessed using histogram charts in addition to central tendency and dispersion measures. The comparison of categorical variables between the three BMI groups was performed using the chi-squared test. Normally distributed continuous variables were compared using one-way analysis of variance (ANOVA), and skewed distributed variables were compared using Kruskal‒Wallis tests. The adjusted and unadjusted effects of HDL-C level on mortality from all causes and MACCEs (the first event of MACCE composite was entered for analyses) were obtained using Cox proportional hazard (CPH) model in groups of HDL ≤ 40, 40 < HDL < 60 (referent), and HDL ≥ 60. Furthermore, Kaplan‒Meier (KM) curves were generated to estimate the cumulative incidence of mortality and MACCE in each BMI-HDL category. The models were adjusted for potential confounders, including age, sex, graft number, diabetes mellitus, hypertension, positive family history (IHD), opium consumption, cigarette smoking, LDL, off-pump CABG, previous MI (within 21 days of surgery), and COPD. The restricted cubic splines (RCS) in the Cox model allow prospecting for a nonlinear relationship of HDL-C level with the “hazard ratio” (HR) of mortality from all causes and MACCEs, estimated from the Cox regression model adjusted for all possible confounders. The RCS analyses were scaled such that the HDL-C level of 40 mg/dL was associated with a hazard ratio of 1. Five knots (df = 4) were applied at the 5^th^, 25^th^, 50^th^, 75^th^, and 95^th^ percentiles of HDL-C levels.

According to the scaled Schoenfeld residuals (for all variables), the proportional hazard assumption was tested. All statistical analyses were conducted by applying R version 4.0.3. Loss-to-

## Results

### Baseline characteristics

**Fig. 1:**
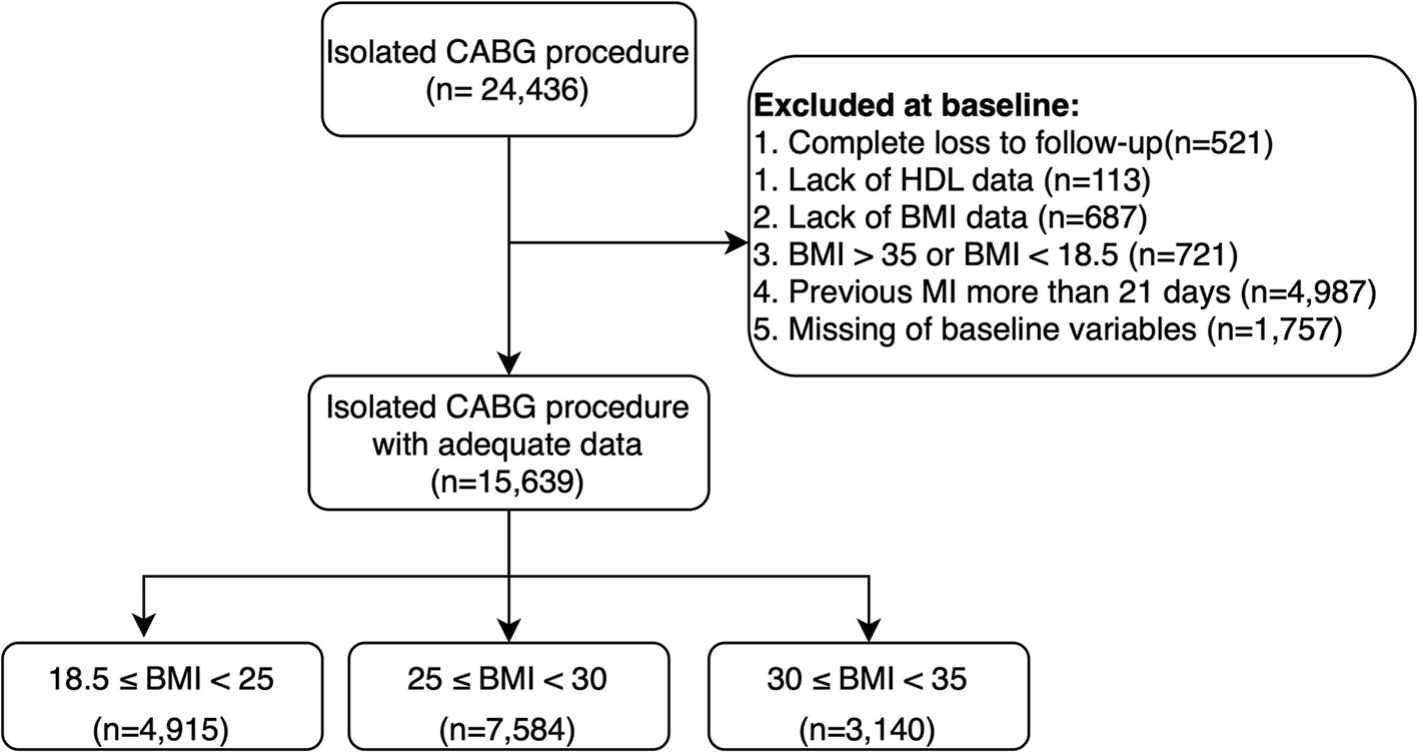
Flow chart of the study cohort

In this registry-based cohort study, 15,639 patients were included (78.08% male, 66.48 years). The flow chart of the study cohort is depicted in Fig. 1. The baseline characteristics of patients according to BMI classification are reported in Table 1. In general, obese patients (30 ≤ BMI < 35) were slightly younger than the normal (18.5 ≤ BMI < 25) and overweight (25 ≤ BMI < 30) groups (65.60 ± 9.91, 66.11 ± 9.91, 67.63 ± 10.12, respectively (*P* < 0.001)). The 30 ≤ BMI < 35 kg/m^2^ group tended to have a higher proportion of diabetes, hypertension, dyslipidemia, and a family history of IHD (*P* < 0.05), while patients with 18.5 ≤ BMI < 25 kg/m^2^ were more likely to smoke cigarettes or use opium (*P* < 0.001).

**Table 1:** Baseline characteristics of the study population (*n* = 15,639)

	18.5 ≤ BMI < 25 (*n* = 4915)	25 ≤ BMI < 30 (*n* = 7584)	30 ≤ BMI < 35 (*n* = 3140)	*P* value
Male sex	3,990 (81.2%)	5914 (75.3%)	2308 (56.4%)	< 0.001
Age (years)	67.63 ± 10.12	66.11 ± 9.91	65.60 ± 9.91	< 0.001
Hypertension	2305 (46.9%)	4225 (53.8%)	2057 (65.5%)	< 0.001
Diabetes	1923 (39.1%)	3110 (41.0%)	1350 (43.5%)	< 0.001
Dyslipidemia	2548 (51.8%)	4381 (57.8%)	2057 (66.5%)	< 0.001
**Smoking status**				< 0.001
Current smoker	1017 (20.7%)	1290 (16.9%)	465 (14.8%)	
Former smoker	870 (17.7%)	1297 (17.1%)	498 (15.8%)	
**Opium Consumption**				< 0.001
Current abuser	664 (13.5%)	883 (11.3%)	298 (9.5%)	
Former abuser	142 (2.9%)	222 (2.8%)	75 (2.4%)	
**Previous MI**				< 0.001
< 24 h	189 (3.6%)	253 (3.2%)	116 (2.8%)	
1–7 days	374 (7%)	524 (6.7%)	226 (5.5%)	
8–21 days	395 (7.4%)	517 (6.6%)	236 (5.8%)	
> 21 days	0 (0%)	0 (0%)	0 (0%)	
**Carotid Stenosis**				0.308
25–50%	28 (0.6%)	29 (0.5%)	18 (0.6%)	
51–75%	19 (0.4%)	17 (0.3%)	7 (0.2%)	
> 75%	38 (0.8%)	59 (1%)	32 (1.1%)	
Graft numbers	3 (3–4)	3 (3–4)	3 (3–4)	< 0.001
COPD	172 (3.5%)	251 (3.3%)	121 (3.9%)	0.191
Cerebrovascular accident	304 (6.2%)	474 (6.2%)	188 (6.0%)	0.321
Family History of IHD	1812 (33.9%)	2863 (36.4%)	1683 (41.3%)	< 0.001
HDL-C (mg/dl)	37 (37–38)	36 (36–37)	37 (37–38)	< 0.001
LDL-C (mg/dl)	89 (88–90)	90 (90–91)	92 (91–94)	< 0.001
Ejection Fraction	47.19 ± 9.21	47.93 ± 8.69	48.61 ± 8.12	< 0.001
eGFR	69.60 ± 21.89	80.32 ± 25.14	94.46 ± 31.43	< 0.001

Data are presented as number (%), mean ± SD, or median (95% confidence interval)

*BMI* Body-Mass Index, *MI* Myocardial Infarction, *COPD* Chronic Obstructive Pulmonary Disease, *IHD* Ischemic heart disease, *HDL-C* High-Density Lipoprotein Cholesterol, *LDL-C* Low–Density Lipoprotein Cholesterol, *eGFR* Estimated Glomerular Filtration Rate

### Endpoints

Among the study cohort, 142 (0.9%) and 1752 (11.2%) patients died at the hospital and later after discharge, respectively. Additionally, regarding MACCEs (first event), 1561 (10.0%), 471 (3.0%), 1901 (12.2%), and 22 (0.1%) experienced ACS, CVA, mortality, and revascularization, respectively. Details on events within each HDL-C and BMI category can be found in Supplementary Table 1.

### HDL-C and mortality among BMI subgroups

**Fig. 2:**
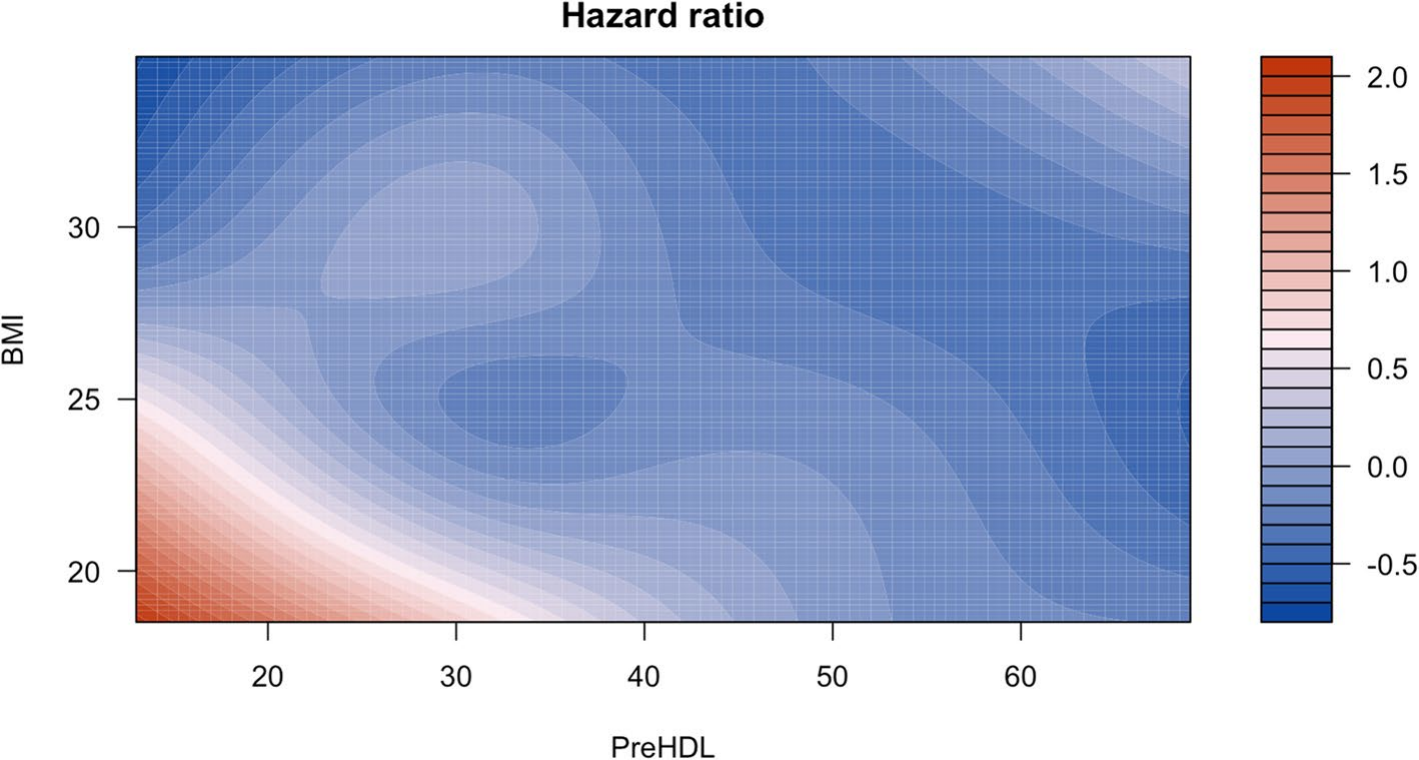
Two-dimensional contour map of BMI effects on HDL-C and all-cause mortality association

Figure 2 displays the adjusted mortality risk in the continuous spectrum of BMI and HDL-C across the entire study cohort, revealing that the highest risk is related to the lowest HDL-C values, especially at lower BMI levels. Interestingly, it is seen that as BMI approaches 30 or more, the expected continuous trend in risk reduction with elevation of HDL-C values (from paler to bolder blue colors), which is present in lower BMI levels, was no longer observed with the highest HDL-C values (evident by turning the bold blue color to pale).

**Fig. 3:**
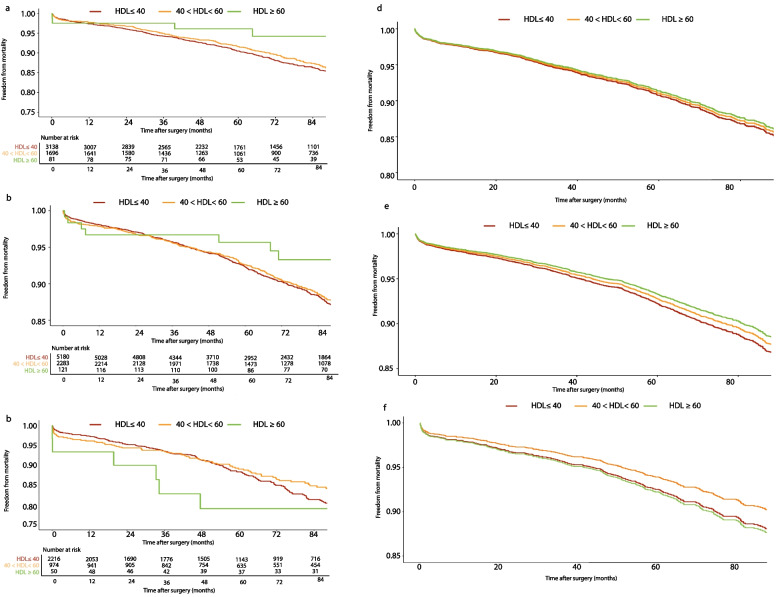
Kaplan‒Meier curves of mortality across the HDL spectrum among different BMI categories **a** Unadjusted- 18.5 ≤ BMI < 25 **b** Unadjusted 25 ≤ BMI < 30 **c** Unadjusted- 30 ≤ BMI < 35 **d** Adjusted- 18.5 ≤ BMI < 25 **e** Adjusted 25 ≤ BMI < 30 **f** Adjusted- 30 ≤ BMI < 35

Table 2 outlines the relationship between categorical HDL-C and study endpoints (mortality and MACCE) using the CPH model. HDL-C ≥ 60 significantly reduced the adjusted all-cause mortality risk (next to 40 < HDL-C < 60) among patients with 18.5 ≤ BMI < 25 and 25 ≤ BMI < 30 (adjusted hazard ratio (aHR): 0.471 (0.242–0.919), *P*: 0.027; and aHR: 0.642 (0.383- 0.981), *P* = 0.007, respectively). The significant protective effect of HDL-C ≥ 60 was no longer observed among the 30 ≤ BMI < 35 groups (aHR: 1.159 (0.564–2.384), *P* = 0.688). Likewise, Supplementary Table 1 indicates that the rate of mortality after discharge was considerably reduced with HDL-C ≥ 60 among 18.5 < BMI < 25 (from 12% in HDL-C ≤ 40 to 6.8% in HDL-C ≥ 60). This improvement was attenuated in the 25 ≤ BMI < 30 group (from 10.6% in HDL-C ≤ 40 to 8.2% in HDL-C ≥ 60) and was no longer observed in the 30 ≤ BMI < 35 group (10.1% in HDL-C ≤ 40 and 9.7% in HDL-C ≥ 60). Figure 3 depicts the KM curves of mortality among various BMI groups at the adjusted and unadjusted levels. Among the normal and overweight patients, those with HDL ≥ 60 had a higher survival rate than the other HDL groups, while this protective effect of HDL ≥ 60 did not hold for obese patients. These findings were in line with the adjusted model on mortality as well.

**Table 2:** HDL-C effects among different BMI subgroups on all-cause mortality and MACCE

**HDL**	Crude				Adjusted			
	Mortality HR (95%CI)	*P* value	MACCE HR (95%CI)	*P* value	Mortality HR (95%CI)	*P* value	MACCE HR (95%CI)	*P* value
**18.5 ≤ BMI < 25 (normal)**								
HDL < 40	1.032 (0.882, 1.209)	0.692	1.150 (1.024, 1.290)	0.018	0.988 (0.842, 1.160)	0.885	1.164 (1.035, 1.310)	0.012
40 < HDL < 60	Reference		Reference		Reference		Reference	
HDL > 60	0.537 (0.276, 1.046)	0.068	0.916 (0.623, 1.346)	0.655	0.471 (0.242, 0.919)	0.027	0.850 (0.578, 1.251)	0.410
**25 ≤ BMI < 30 (overweight)**								
HDL < 40	1.013 (0.884, 1.161)	0.854	1.079 (0.981, 1.186)	0.119	0.999 (0.868, 1.150)	0.986	1.084 (0.983, 1.196)	0.106
40 < HDL < 60	Reference		Reference		Reference		Reference	
HDL > 60	0.683 (0.407, 1.145)	0.148	0.858 (0.618, 1.190)	0.358	0.642 (0.383, 0.981)	0.007	0.835 (0.601, 1.159)	0.281
**30 ≤ BMI < 35 (obese)**								
HDL < 40	1.225 (0.974, 1.539	0.082	1.057 (0.914, 1.222)	0.456	1.205 (0.981, 1.530)	0.081	1.123 (1.021, 1.278)	0.012
40 < HDL < 60	Reference		Reference		Reference		Reference	
HDL > 60	1.063 (0.519, 2.178)	0.867	0.896 (0.549, 1.462)	0.661	1.159 (0.564, 2.384)	0.688	0.906 (0.555, 1.481)	0.695

*HDL-C* High-density lipoprotein cholesterol, *BMI* Body mass index, *MACCE* Major adverse cardio-cerebrovascular events

### RCS trend analyses on HDL-C and mortality among BMI subgroups

**Fig. 4:**
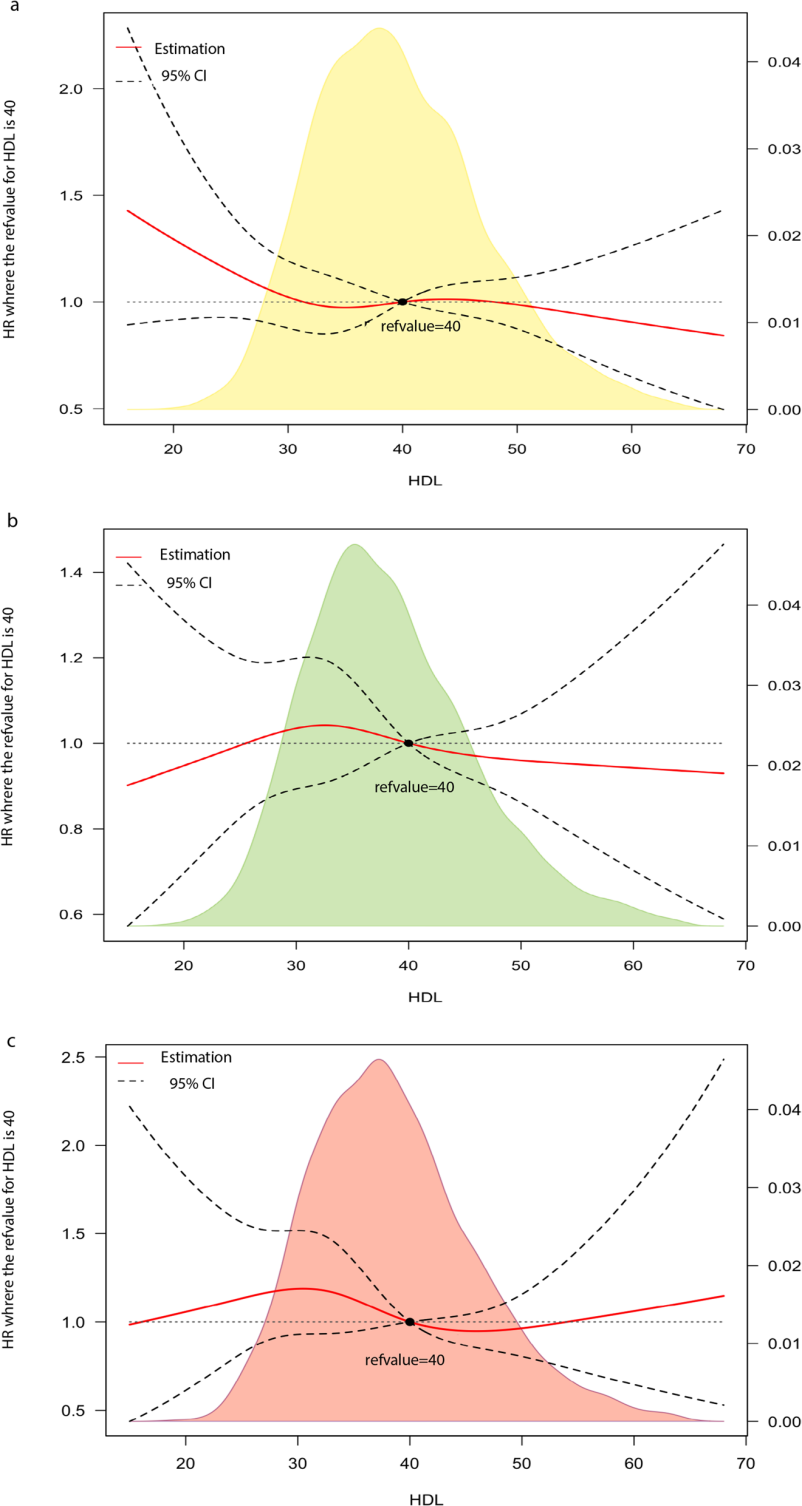
Restricted cubic spline plots on HDL-C and all-cause mortality **a** 18.5 ≤ BMI < 25 **b** 35 ≤ BMI < 30 **c** 30 ≤ BMI < 35

To evaluate the trend of HDL-C effects, RCS analyses were applied. The RCS graph, in line with the CPH model (Fig. 4), revealed an overall descending trend in the adjusted risk of mortality from all causes (with minuscule fluctuation around HDL-C = 40) in 18.5 ≤ BMI < 25, with the majority of the HDL-C spectrum showing a linear inverse association. Among 25 ≤ BMI < 30, there was also a linear decline from HDL-C≈35 onward. Nevertheless, among 30 ≤ BMI < 35, no uniform inverse linear relationship was observed; patients with HDL-C values < 40 had an increased mortality risk; after HDL-C = 40, the risk fell below 1 (aHR < 1), while no further risk reduction was observed after HDL-C≈55 (aHR > 1).

### HDL-C and MACCE among BMI subgroups

**Fig. 5:**
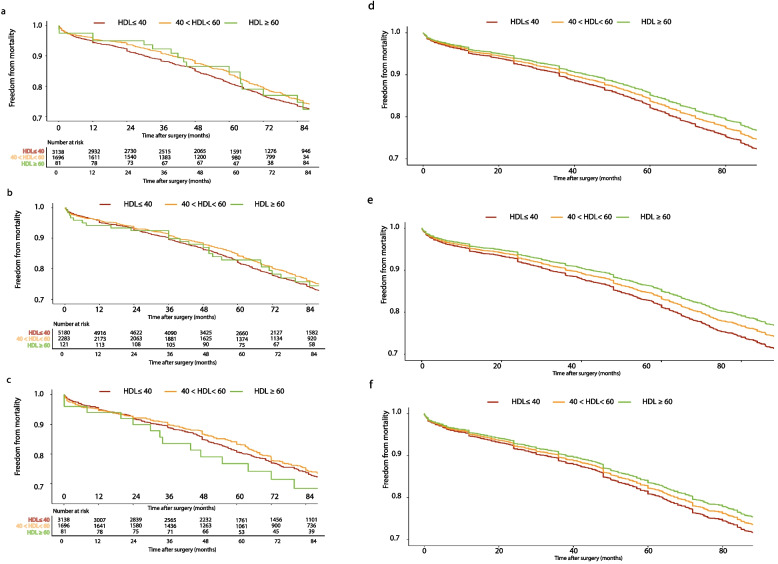
Kaplan‒Meier curves of MACCEs across the HDL spectrum among different BMI categories **a** Unadjusted- 18.5 ≤ BMI < 25 **b** Unadjusted 25 ≤ BMI < 30 **c** Unadjusted- 30 ≤ BMI < 35 **d** Adjusted- 18.5 ≤ BMI < 25 **e** Adjusted 25 ≤ BMI < 30 **f** Adjusted- 30 ≤ BMI < 35

CPH models on MACCE composite (Table 2) revealed a significant or near significant hazardous effect of HDL-C < 40 in all BMI categories (normal: aHR: 1.164 (1.035–1.310), *P*: 0.012; overweight: aHR: 1.084 (0.983–1.196), *P*: 0.106; obese: aHR: 1.123 (1.021–1.278), *P*: 0.012). Figure 5 depicts the KM curves of MACCEs among various BMI groups at the adjusted and unadjusted levels. Freedom from MACCE events in different HDL groups was more similar between BMI categories, with HDL < 40 demonstrating the highest MACCE events. These findings were consistent with the adjusted KM curves of MACCEs.

### RCS trend analyses on HDL-C and MACCE among BMI subgroups

**Fig. 6:**
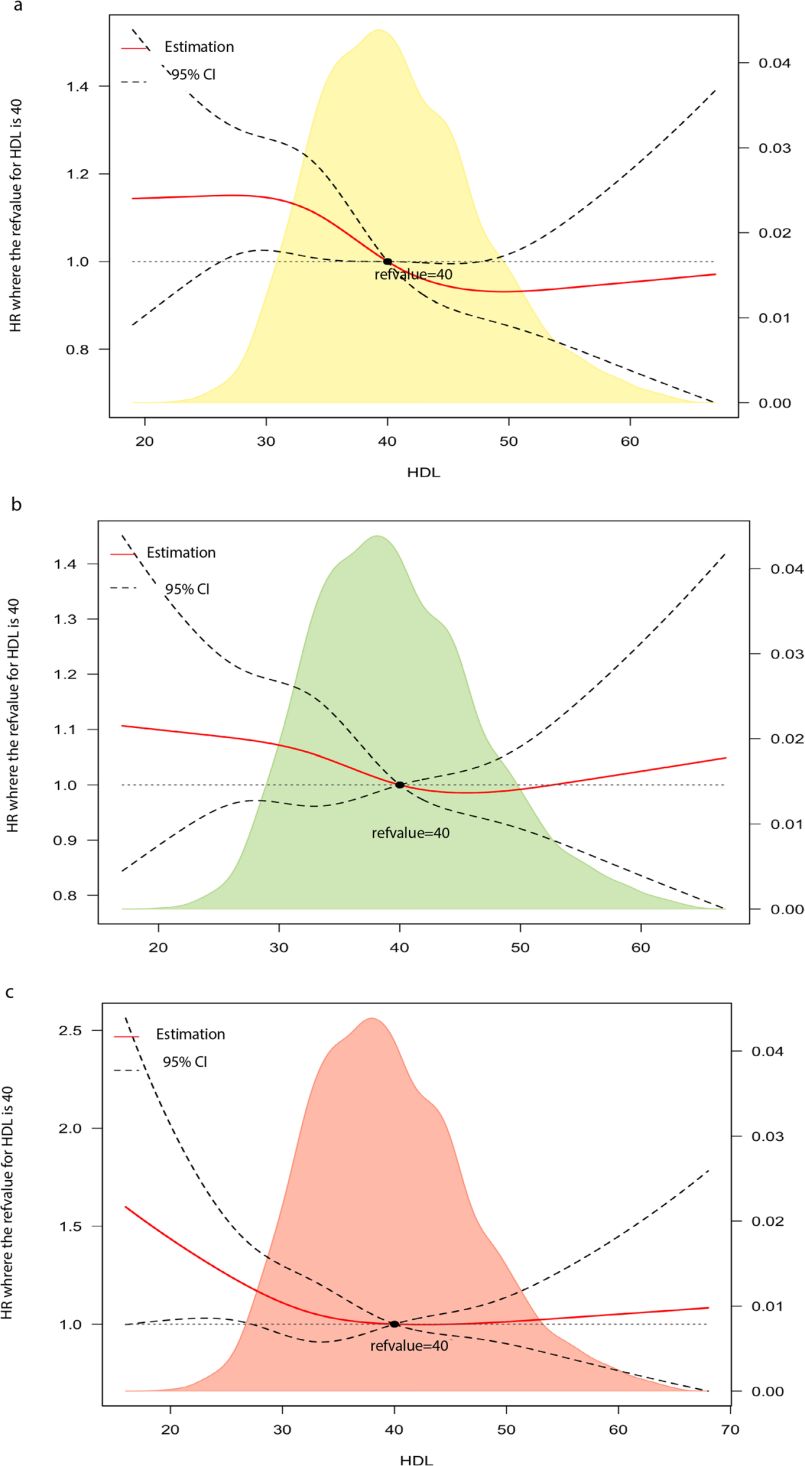
Restricted cubic spline plots on HDL-C and MACCE **a** 18.5 ≤ BMI < 25 **b** 35 ≤ BMI < 30 **c** 30 ≤ BMI < 35

RCS analyses (Fig. 6) did not show an inverse and uniform relationship between HDL-C and MACCEs in any BMI category. In normal BMI patients, HDL-C > 40 was protective (MACCE aHR < 1) (compared to HDL-C = 40), while there was a plateau effect followed by a modest increment in the overweight-obese population.

## Discussion

The current prospective registry-based study evaluated the possibility of whether BMI affects the relationship between serum HDL-C levels and CABG patient outcomes. This study showed that the association between HDL-C and poorer results after CABG was nonlinear among the highest BMI spectrum. CPH analyses revealed that although HDL-C ≥ 60 exerted protective effects on all-cause mortality among patients with BMI < 30, its protective effect was no longer observed among patients with 30 ≤ BMI < 35. RCS analyses recapitulated these findings. Furthermore, RCS graphs revealed that HDL-C was associated with MACCEs in a nonlinear manner among overweight-obese patients, where the expected risk reduction was not observed from HDL-C≈55 onward. These findings suggest that values at the top of the HDL-C cholesterol spectrum were not associated with better outcomes among obese patients after CABG.

### Comparisons with other studies- what does the current work add to the existing knowledge?

It has only been in recent years that the expected cardiovascular risk reduction with HDL elevation has been challenged [20–26]. Regarding the significance and novelty of this subject, one of the key messages of the 2016 European Society Guideline (ESC) for Cardiovascular Disease Prevention [20] was that although low HDL-C increased cardiovascular risk, HDL-increasing measures were not proven to reduce the risk. Similarly, ESC 2021 indicated that a very high HDL-C level might be a sign of an increase in cardiovascular risk [21].

Furthermore, despite the inextricable link between BMI and various aspects of HDL pathophysiology (as discussed above), studies that measure the effect of this link on cardiovascular outcomes are scarce. These issues prompted us to try to narrow this knowledge gap as much as possible with the resources at our disposal. Nevertheless, clinical studies and Mendelian randomizations are warranted to elucidate the role of HDL in cardiovascular risk assessment. Since, to date, no therapeutic goal has been defined for HDL-C from clinical trials [21], well-designed observational studies can pave the way for performing such studies.

### Nonlinear association between HDL-C and cardiovascular outcomes

Previous observations have reported that HDL-C concentrations affect CAD outcomes independently in an inverse and linear fashion [27, 28]. However, these conventional implications of high HDL-C levels have been debated in more recently published studies, reporting a U-shaped association of HDL-C levels with mortality and cardiovascular events [22–26, 29]. Among the first studies that changed the perception of HDL was a Mendelian randomized study published in 2012, revealing that not all HDL-C-increasing-associated genetic mechanisms diminish the risk of MI [29]. Other studies have reported poorer outcomes in very high levels of HDL-C [22–24]. The exact threshold of very high serum HDL-C levels varies among different studies. However, recent findings from a pooled analysis (37 prospective cohort studies) across the general population with very high HDL-C demonstrated a J-shaped pattern in terms of mortality risk reduction from all, cardiovascular, and cancer-related causes, with no further risk reduction from HDL-C ≥ 77 onward [25]. A meta-analysis of cohort studies (more than 43 thousand patients) revealed that HDL-C > 90 more than doubled the mortality risk of atherosclerotic cardiovascular disease [26]. The most recently published study by Chang Liu et al. reported that HDL-C levels > 80 were unexpectedly related to worsened outcomes in individuals with CAD compared to 40 < HDL-C < 60. Indeed, HDL-C > 80 increased the adjusted overall mortality risk by 96% for all causes and 71% for cardiovascular death. They also demonstrated a U-shaped association between HDL-C and poorer outcomes, whereby the highest risks were observed at the highest and lowest values in the HDL-C spectrum [30].

Kaur et al. found that 30 < HDL-C < 50 mg/dL correlated with the most favorable outcomes, while HDL-C levels out of this range were linked to an augmented risk of mortality (not MACCE) in men undergoing percutaneous coronary intervention (PCI) [8]. Angeloni et al.’s survey focused on the CABG population. They concluded that very high preoperative HDL-C levels enhanced MACCE occurrence during follow-ups [31]. The inconsistencies in determining the exact value of the so-called “high HDL-C levels” are reasonably justifiable based on various types of the study population (patients with CAD, PCI, CABG), diets (for example, the amount of fish and nut consumption), exercise level, races and ethnicities, and the rate plus dose of statin use.

### BMI modifies the HDL-C effect on the cardiovascular system

To date, little evidence is available concerning the effect of BMI on the correlation between HDL-C levels and cardiovascular prognosis. In this regard, De Lima-Junior et al. attempted to investigate the relationship between the antiatherosclerotic function of HDL-C and BMI in 899 asymptomatic patients. They reported that individuals with increased HDL-C experienced an attenuated increase in carotid intima-media thickness (cIMT) with BMI elevation (interaction effect ß = -0.054). Furthermore, BMI was inversely related to HDL-C activity in inhibiting platelet aggregation and cholesterol efflux capacity. Taken together, this study suggested that the increase in BMI was linked to impaired cardioprotective functions of HDL-C and a concomitant increase in atherosclerosis [32]. Furthermore, an inverse correlation between serum HDL-C concentration and stroke has been reported among those with BMI < 24 kg/m^2^; however, this finding no longer proved true for patients with BMI ≥ 24 kg/m^2^ [33].

Possible explanations for these findings will be reviewed here: 1) Obese patients had more cardiovascular risk factors, which might have neutralized the advantageous effect of higher HDL-C. However, the adjusted results did not prove this statement. Dysfunction of HDL-C has been identified in metabolic (diabetes, CKD, and metabolic syndrome) and autoimmune conditions. Thus, it is no surprise that elevated HDL-C in some selected patients could be related to a higher risk of CADs and mortality [34]. The lack of anti-inflammatory properties of HDL-C was shown in patients with stable or unstable CAD via reduced paraoxonase 1 (PON1) activity, which leads to inhibition of endothelial NOS activation [35].

2) The function of HDL-C may become impaired in obesity. While there are no solid underlying mechanisms for the inverse relationship between very high HDL-C levels and outcomes in obesity, emerging evidence has elucidated that obesity status can exert several effects on HDL-C. These implications include HDL-C metabolism, functionality, and subclass distribution, particularly reducing cholesterol efflux, which has a more important independent impact on the development of CADs [36, 37]. Obesity is associated with deregulation of metabolism in hypertrophic adipose tissue, which causes the activation of unusual amounts of reactive oxygen species and oxidative stress. As a result, these processes contribute to the activation of systemic inflammatory pathways [38]. The loss of protective characteristics of HDL-C in obesity may refer to chronic, low-grade adipocyte inflammation, as seen in the obesity status, and accumulation of macrophages in the adipose tissue. As a result, proinflammatory mediators, in particular TNFα, are produced more extensively [16]. Adipocytes control cholesterol efflux by recruiting scavenger receptor class B type I (SR-BI) and ATP-binding cassette transporter A1 (ABCA1). Furthermore, it has been found that TNF-α could impair cholesterol efflux of HDL-C by downregulating ABCA1 and SR-BI expression [17, 39]. In addition, some conditions, such as diabetes, obesity, and inflammation, could damage Apo A-I. This protein constitutes a major component of HDL-C particles and is essential for the reverse cholesterol transporting (RCT) role of HDL-C via oxidative mechanisms. Thus, the RCT ability of HDL-C is reduced, leading to dysfunction and proinflammatory effects of the HDL-C molecule [39, 40]. Furthermore, the ability of HDL-C to neutralize the inhibitory effects of oxidized LDL-C on endothelial-related vasodilation was abolished in abdominally obese subjects [41].

3) Another possible underlying cause of adverse effects of very high HDL-C concentrations in obese patients centers around serum visfatin, an adipocytokine with insulin-mimetic properties. Several studies have demonstrated that serum visfatin levels increase in obese patients and correlate with HDL-C positively, while this correlation does not seem to exist in their nonobese counterparts. Nevertheless, the exact biological mechanisms of the relationship between serum visfatin and HDL-C remain obscure [42, 43]. It has also been indicated that visfatin may be crucial in inflammatory processes in overweight patients [44]. Regarding the inflammatory role of visfatin, prior studies have suggested that visfatin might localize to foam cell macrophages within unstable atherosclerotic plaques, resulting in plaque destabilization/rupture [45]. Under inflammatory conditions, HDL-C may lose its anti-inflammatory and antioxidative properties while also gaining proinflammatory functions, thus impairing the antiatherogenic effects of HDL-C. As such, HDL-C tends to convert to a proinflammatory agent and thus could increase the risk of CAD by activating inflammatory pathways [46]. Consistently, several studies have reported that during the acute phase response, HDL-C could become a proinflammatory and proatherogenic molecule, which is mediated by some proteins, including serum amyloid A (SAA) and ceruloplasmin, binding to HDL-C molecules. As a result, these processes may restrict HDL-C’s ability to enhance RCT and preclude LDL modification [34].

4) Another mechanism could be deterioration of HDL-C subclass distribution. A previous study indicated an association between obesity and reduced large HDL-C subfractions [16]. Additionally, it has been demonstrated that decreased amounts of high-molecular weight HDL-C particles and increased amounts of low-molecular weight HDL-C particles were remarkably associated with higher cardiovascular risk [47]. Thus, it might be concluded that extremely increased HDL-C levels may reflect a greater increase in small HDL-C particles among obese individuals, resulting in a worsened prognosis. Furthermore, elevated triglycerides and LDL, which are more frequent among obese populations, can directly interfere with the antiatherogenic action of HDL-C and are inversely related to the vasodilatory activity of HDL-C [39]. Interestingly, bariatric surgery has been suggested to notably improve the structure and function of HDL-C [34]. Ultimately, these results highlighted the role of personalized medicine, suggesting that not the same HDL-C level might be necessarily beneficial to different individuals. In particular, various distinct genetic variants have been reported to affect HDL-C levels and function, as well as statin pharmacokinetics [48].

Accordingly, HDL-C functionality seems to play a far more essential cardioprotective role than circulating HDL-C levels. Furthermore, not only does a very high HDL-C level not offer invariable healthy benefits but also, in some specific states, may turn into a pro-inflammatory agent, thereby increasing cardiovascular risk.

This study highlighted a considerable finding on the role of BMI in the correlation between very high HDL-C levels and worsened outcomes among the CABG population. Altogether, the mechanisms through which highly elevated HDL-C levels correlate with possible poorer cardiovascular outcomes in obese patients remain unclear. Nevertheless, it may have clinical and public health implications when HDL-C cholesterol is applied for risk assessment. Ultimately, further larger sample studies among different ethnicities and populations are warranted to explore the obesity implications on the relationship between HDL-C concentrations and outcomes post-CABG.

### Study strengths and limitations

The inclusion of a large population from the registry system of Tehran Heart Center was the primary strength of the present study. Second, this study evaluated the full range of HDL-C levels in each BMI category using both multivariate Cox regression and RCS analysis. Finally, detailed information on several confounder variables with well-known effects on outcomes after CABG was provided in this study. On the other hand, this study was limited by some issues. First, it was conducted only on the Iranian population. Ethnicity may be associated with HDL-C functionality, thereby restricting the generalizability of the current results to other populations. Another key limitation was omitting the role of lifestyle modifications on HDL-C and weight during follow-up. This study captured the baseline data of the population. In addition, the number of events at the highest HDL-C values was inevitably small, necessitating further exploration of the results in other large-scale studies.

## Conclusions

The present study indicated that no linear inverse relationship existed between higher HDL-C and better outcomes after CABG among obese patients. Indeed, the benefits of higher concentrations of HDL-C did not hold for extremely high concentrations in patients with BMI ≥ 30. This observation could imply heterogeneity in the risk of obese subjects with high HDL-C, suggesting a role of obesity in the function and properties of HDL-C. These observations can be reflected in clinical practice when performing cardiovascular risk assessment and planning treatment strategies, emphasizing that clinicians should consider the entire spectrum of concomitant cardiovascular risk factors together, while not assuming that a very high HDL-C is necessarily and completely protective against poorer outcomes among all CABG patients. Additionally, the nonlinear relationship between HDL-C and poorer outcomes reported in recent studies might be in part justified on the grounds of BMI. Future systematic reviews/meta-analyses can elucidate the answer to some of these questions. Furthermore, these findings suggested that the nonnecessarily protective effects of high HDL-C might not apply to all groups of patients. This matter can guide clinicians and researchers to prospect for at-risk groups.

## Supplementary Information

**Additional file 1: Supplementary Table 1.** Number of outcomes.

## Data Availability

The datasets used and/or analyzed during the current study are available from the corresponding author on reasonable request.

## Abbreviations

BMI: Body mass index
HDL: High-density lipoprotein
CABG: Coronary artery bypass graft
MACCE: Major adverse cardio-cerebrovascular events
ACS: Acute coronary syndrome
CVA: Cerebrovascular accidents
CPH: Cox proportional hazard
RCS: Restricted cubic spline

## References

[1] Brown JC, Gerhardt TE, Kwon E. Risk Factors For Coronary Artery Disease. In: StatPearls. StatPearls Publishing, Treasure Island (FL); 2022. PMID: 32119297.

[2] Habib RH, Dimitrova KR, Badour SA, Yammine MB, El-Hage-Sleiman A-KM, Hoffman DM, et al. CABG versus PCI: greater benefit in long-term outcomes with multiple arterial bypass grafting. Journal of the American College of Cardiology. 2015;66(13):1417–27. 10.1016/j.jacc.2015.07.060 PMC5473156 26403338

[3] Sabik JF, Blackstone EH, Gillinov AM, Smedira NG, Lytle BW (2006). Occurrence and risk factors for reintervention after coronary artery bypass grafting. Circulation..

[4] Yanagawa B, Algarni KD, Singh SK, Deb S, Vincent J, Elituv R (2014). Clinical, biochemical, and genetic predictors of coronary artery bypass graft failure. J Thorac Cardiovasc Surg.

[5] Used R (2012). Lipid-related markers and cardiovascular disease prediction. JAMA.

[6] Goyfman M, Chaus A, Dabbous F, Tamura L, Sandfort V, Brown A, et al. The correlation of dyslipidemia with the extent of coronary artery disease in the multiethnic study of atherosclerosis. J lipids. 2018;2018:5607349. 10.1155/2018/5607349. 10.1155/2018/5607349 PMC5892234 29785308

[7] Arnett DK, Blumenthal RS, Albert MA, Buroker AB, Goldberger ZD, Hahn EJ (2019). 2019 ACC/AHA guideline on the primary prevention of cardiovascular disease: executive summary: a report of the American College of Cardiology/American Heart Association Task Force on Clinical Practice Guidelines. J Am Coll Cardiol.

[8] Kaur M, Ahuja KR, Khubber S, Zhou L, Verma BR, Meenakshisundaram C (2021). Effect of High-Density Lipoprotein Cholesterol Levels on Overall Survival and Major Adverse Cardiovascular and Cerebrovascular Events. Am J Cardiol.

[9] Chen C-l, Liu X-c, Liu L, Lo K, Yu Y-l, Huang J-y, et al. U-Shaped Association of High-Density Lipoprotein Cholesterol with All-Cause and Cardiovascular Mortality in Hypertensive Population. Risk Management and Healthcare Policy. 2020;13:2013. 10.2147/RMHP.S272624 PMC7549655 33116982

[10] Yi SW, Park SJ, Yi JJ, Ohrr H, Kim H (2021). High-density lipoprotein cholesterol and all-cause mortality by sex and age: a prospective cohort study among 15.8 million adults. Int J Epidemiol.

[11] Lavie CJ, De Schutter A, Parto P, Jahangir E, Kokkinos P, Ortega FB (2016). Obesity and prevalence of cardiovascular diseases and prognosis—the obesity paradox updated. Prog Cardiovasc Dis.

[12] Wilson PW, D’Agostino RB, Sullivan L, Parise H, Kannel WB. Overweight and obesity as determinants of cardiovascular risk: the Framingham experience. Arch Intern Med. 2002;162(16):1867–72. 10.1001/archinte.162.16.1867 12196085

[13] Angerås O, Albertsson P, Karason K, Råmunddal T, Matejka G, James S (2013). Evidence for obesity paradox in patients with acute coronary syndromes: a report from the Swedish Coronary Angiography and Angioplasty Registry. Eur Heart J.

[14] Doehner W, Gerstein HC, Ried J, Jung H, Asbrand C, Hess S (2020). Obesity and weight loss are inversely related to mortality and cardiovascular outcome in prediabetes and type 2 diabetes: data from the ORIGIN trial. Eur Heart J.

[15] Masoudkabir F, Yavari N, Jameie M, Pashang M, Sadeghian S, Salarifar M, et al. The association between different body mass index levels and midterm surgical revascularization outcomes. PloS one. 2022;17(9):e0274129. 10.1371/journal.pone.0274129 PMC9522296 36174074

[16] Zhang T, Chen J, Tang X, Luo Q, Xu D, Yu B (2019). Interaction between adipocytes and high-density lipoprotein: new insights into the mechanism of obesity-induced dyslipidemia and atherosclerosis. Lipids Health Dis.

[17] Wang H, Peng D-Q (2011). New insights into the mechanism of low high-density lipoprotein cholesterol in obesity. Lipids Health Dis.

[18] Sheikhy A, Fallahzadeh A, Sadeghian S, Forouzannia K, Bagheri J, Salehi-Omran A (2021). Mid-term outcomes of off-pump versus on-pump coronary artery bypass graft surgery; statistical challenges in comparison. BMC Cardiovasc Disord.

[19] Khalaji A, Behnoush AH, Jameie M, Sharifi A, Sheikhy A, Fallahzadeh A, et al. Machine learning algorithms for predicting mortality after coronary artery bypass grafting. Front Cardiovasc Med. 2022;9:977747. 10.3389/fcvm.2022.977747. 10.3389/fcvm.2022.977747 PMC9448905 36093147

[20] Piepoli MF, Hoes AW, Agewall S, Albus C, Brotons C, Catapano AL (2016). 2016 European Guidelines on cardiovascular disease prevention in clinical practice. Kardiologia Polska (Polish Heart Journal).

[21] Visseren FL, Mach F, Smulders YM, Carballo D, Koskinas KC, Bäck M (2021). 2021 ESC Guidelines on cardiovascular disease prevention in clinical practice: Developed by the Task Force for cardiovascular disease prevention in clinical practice with representatives of the European Society of Cardiology and 12 medical societies With the special contribution of the European Association of Preventive Cardiology (EAPC). Eur Heart J.

[22] Madsen CM, Varbo A, Nordestgaard BG (2017). Extreme high high-density lipoprotein cholesterol is paradoxically associated with high mortality in men and women: two prospective cohort studies. Eur Heart J.

[23] Madsen CM, Varbo A, Nordestgaard BG (2021). Novel insights from human studies on the role of high-density lipoprotein in mortality and noncardiovascular disease. Arterioscler Thromb Vasc Biol.

[24] Han BH, Han K, Yoon KH, Kim MK, Lee SH (2020). Impact of Mean and Variability of High-Density Lipoprotein-Cholesterol on the Risk of Myocardial Infarction, Stroke, and Mortality in the General Population. J Am Heart Assoc.

[25] Zhong GC, Huang SQ, Peng Y, Wan L, Wu YQL, Hu TY (2020). HDL-C is associated with mortality from all causes, cardiovascular disease and cancer in a J-shaped dose-response fashion: a pooled analysis of 37 prospective cohort studies. Eur J Prev Cardiol.

[26] Hirata A, Sugiyama D, Watanabe M, Tamakoshi A, Iso H, Kotani K, et al. Association of extremely high levels of high-density lipoprotein cholesterol with cardiovascular mortality in a pooled analysis of 9 cohort studies including 43,407 individuals: The EPOCH–JAPAN study. Journal of clinical lipidology. 2018;12(3):674–84. e5. 10.1016/j.jacl.2018.01.014 29506864

[27] Ko DT, Alter DA, Guo H, Koh M, Lau G, Austin PC (2016). High-density lipoprotein cholesterol and cause-specific mortality in individuals without previous cardiovascular conditions: the CANHEART study. J Am Coll Cardiol.

[28] Gordon DJ, Probstfield JL, Garrison RJ, Neaton JD, Castelli WP, Knoke JD (1989). High-density lipoprotein cholesterol and cardiovascular disease. Four prospective American studies Circulation.

[29] Voight BF, Peloso GM, Orho-Melander M, Frikke-Schmidt R, Barbalic M, Jensen MK (2012). Plasma HDL cholesterol and risk of myocardial infarction: a mendelian randomisation study. The Lancet.

[30] Liu C, Dhindsa D, Almuwaqqat Z, Ko Y-A, Mehta A, Alkhoder AA, et al. Association Between High-Density Lipoprotein Cholesterol Levels and Adverse Cardiovascular Outcomes in High-risk Populations. JAMA Cardiol. 2022;7(7):672–80. 10.1001/jamacardio.2022.0912 PMC9118072 35583863

[31] Angeloni E, Paneni F, Landmesser U, Benedetto U, Melina G, Lüscher TF (2013). Lack of protective role of HDL-C in patients with coronary artery disease undergoing elective coronary artery bypass grafting. Eur Heart J.

[32] de Lima-Junior JC, Virginio VW, Moura FA, Bertolami A, Bertolami M, Coelho-Filho OR (2020). Excess weight mediates changes in HDL pool that reduce cholesterol efflux capacity and increase antioxidant activity. Nutr Metab Cardiovasc Dis.

[33] Yu Y, Hu L, Huang X, Zhou W, Bao H, Cheng X (2021). BMI modifies the association between serum HDL cholesterol and stroke in a hypertensive population without atrial fibrillation. J Endocrinol Invest.

[34] Kosmas CE, Martinez I, Sourlas A, Bouza KV, Campos FN, Torres V, et al. High-density lipoprotein (HDL) functionality and its relevance to atherosclerotic cardiovascular disease. Drugs in context. 2018;7:212525. 10.7573/dic.212525 PMC5877920 29623098

[35] Besler C, Heinrich K, Rohrer L, Doerries C, Riwanto M, Shih DM (2011). Mechanisms underlying adverse effects of HDL on eNOS-activating pathways in patients with coronary artery disease. J Clin Investig.

[36] McGillicuddy FC, Reilly MP, Rader DJ (2011). Adipose modulation of high-density lipoprotein cholesterol: implications for obesity, high-density lipoprotein metabolism, and cardiovascular disease. Am Heart Assoc..

[37] Borggreve S, De Vries R, Dullaart R (2003). Alterations in high-density lipoprotein metabolism and reverse cholesterol transport in insulin resistance and type 2 diabetes mellitus: role of lipolytic enzymes, lecithin: cholesterol acyltransferase and lipid transfer proteins. Eur J Clin Invest.

[38] Monteiro R, Azevedo I. Chronic inflammation in obesity and the metabolic syndrome. Mediators Inflamm. 2010;2010. 10.1155/2010/289645 PMC2913796 20706689

[39] Stadler JT, Marsche G (2020). Obesity-related changes in high-density lipoprotein metabolism and function. Int J Mol Sci.

[40] Sacks FM, Jensen MK (2018). From high-density lipoprotein cholesterol to measurements of function: prospects for the development of tests for high-density lipoprotein functionality in cardiovascular disease. Arterioscler Thromb Vasc Biol.

[41] Perségol L, Vergès B, Gambert P, Duvillard L (2007). Inability of HDL from abdominally obese subjects to counteract the inhibitory effect of oxidized LDL on vasorelaxation. J Lipid Res.

[42] Johansson LM, Johansson LE, Ridderstråle M (2008). The visfatin (PBEF1) G-948T gene polymorphism is associated with increased high-density lipoprotein cholesterol in obese subjects. Metabolism.

[43] Jin H, Jiang B, Tang J, Lu W, Wang W, Zhou L (2008). Serum visfatin concentrations in obese adolescents and its correlation with age and high-density lipoprotein cholesterol. Diabetes Res Clin Pract.

[44] Zhang YY, Gottardo L, Thompson R, Powers C, Nolan D, Duffy J (2006). A visfatin promoter polymorphism is associated with low-grade inflammation and type 2 diabetes. Obesity.

[45] Dahl TB, Yndestad A, Skjelland M, Øie E, Dahl A, Michelsen A (2007). Increased expression of visfatin in macrophages of human unstable carotid and coronary atherosclerosis: possible role in inflammation and plaque destabilization. Circulation.

[46] Rosenson RS, Brewer HB, Ansell BJ, Barter P, Chapman MJ, Heinecke JW (2016). Dysfunctional HDL and atherosclerotic cardiovascular disease. Nat Rev Cardiol.

[47] Kontush A, Chapman MJ (2006). Antiatherogenic small, dense HDL—guardian angel of the arterial wall?. Nat Clin Pract Cardiovasc Med.

[48] Sheikhy A, Fallahzadeh A, Aghaei Meybodi HR, Hasanzad M, Tajdini M, Hosseini K (2021). Personalized medicine in cardiovascular disease: review of literature. J Diabetes Metab Disord.

