# Supplementary material for: BMI modifies HDL-C effects on coronary artery bypass grafting outcomes
Source: Lipids Health Dis. 2022 Nov 29;21:128. doi: 10.1186/s12944-022-01739-2 (PMC9710033; doi:10.1186/s12944-022-01739-2)
Supplement: Supplementary file 1 — Additional file 1: Supplementary Table 1. Number of outcomes. [file 12944_2022_1739_MOESM1_ESM.docx]

| Supplementary table 1. Number of outcomes | | | | | | | | |
| --- | --- | --- | --- | --- | --- | --- | --- | --- |
| Outcome |  | | All-cause mortality | | MACCE (first event) | | | |
| Group |  | | In hospital | After discharge | ACS | CVA | All-cause mortality | revascularization |
| Overall cohort (n=15639) | | | | | | | | |
|  | | | 142 (0.9%) | 1752 (11.2%) | 1561  (10.0%) | 471  (3.0%) | 1901 (12.2%) | 22  (0.1%) |
| HDL groups | | | | | | | | |
| HDL =< 40  (n=10434) | | | 78 (0.8%) | 1076 (10.9%) | 955  (9.7%) | 311 (3.2%) | 1158 (11.7%) | 13  (0.1%) |
| 40<HDL<60  (n=2894) | | | 57 (1.0%) | 651 (11.9%) | 567 (10.4%) | 149 (2.7%) | 710 (13.0%) | 9  (0.2%) |
| HDL >= 60 (n=311) | | | 7 (2.3%) | 25  (8.0%) | 39  (12.5%) | 11 (3.5%) | 33 (10.6%) | 0 |
| BMI and HDL groups | | | | | | | | |
| 18.5 < BMI < 25  (n=4915) | | HDL =< 40  (n=3138) | 28 (0.9%) | 356 (12.0%) | 279  (9.4%) | 88 (3.0%) | 385 (12.9%) | 2  (0.1%) |
|  |  | 40<HDL<60  (n=1674) | 16 (0.9%) | 245 (13.3%) | 164  (8.9%) | 46 (2.5%) | 262 (14.3%) | 3  (0.2%) |
|  |  | HDL >= 60  (n=103) | 2 (1.9%) | 7  (6.8 %) | 11  (10.7%) | 6 (5.8%) | 11 (10.7%) | 0 |
| 25 <= BMI < 30  (n=7584) | | HDL =< 40  (n=5180) | 35 (0.7%) | 519 (10.6%) | 468  (9.6%) | 150 (3.1%) | 567 (11.6%) | 7  (0.1%) |
|  |  | 40<HDL<60  (n=2258) | 25 (1.0%) | 309 (12.2%) | 259 (10.2%) | 73 (2.9%) | 335 (13.2%) | 2  (0.1%) |
|  |  | HDL >= 60  (n=146) | 3 (2.1%) | 12  (8.2%) | 22  (15.1%) | 2 (1.4%) | 14  (9.6%) | 0 |
| 30 <= BMI < 35  (n=3140) | | HDL =< 40  (n=2216) | 15 (0.8% | 201 (10.1%) | 208 (10.4%) | 73 (3.7%) | 206 (10.3%) | 4  (0.2%) |
|  |  | 40<HDL<60  (n=962) | 16 (1.5%) | 97  (9.0%) | 144 (13.3%) | 30 (2.8%) | 113 (10.4%) | 4  (0.4%) |
|  |  | HDL >= 60  (n=62) | 2 (3.2%) | 6  (9.7%) | 6  (9.7%) | 3 (4.8%) | 8  (12.9%) | 0 |

*MACCE: major adverse cardio-cerebrovascular events; ACS: acute coronary syndrome, CVA: cerebrovascular accidents, HDL: high-density lipoprotein, BMI: body mass index
